# Sciatic Nerve Stimulation Mitigates Depression‐Like Behaviors and Memory Deficits in Stressed Mice

**DOI:** 10.1002/kjm2.70091

**Published:** 2025-08-26

**Authors:** Chih‐Hao Tien, Pei‐Wen Chen, Ya‐Hsin Hsiao, Chia‐En Wong, Ming‐Tse Wu, Ying‐Fei Chen, Kuo‐Chang Huang, Po‐Hsuan Lee, Kun‐Chia Chang, Heng‐Juei Hsu, Jung‐Shun Lee

**Affiliations:** ^1^ Division of Neurosurgery, Department of Surgery National Cheng Kung University Hospital Tainan Taiwan; ^2^ Department of Cell Biology and Anatomy, College of Medicine National Cheng Kung University Tainan Taiwan; ^3^ Department of Pharmacology, College of Medicine National Cheng Kung University Tainan Taiwan; ^4^ Institute of Basic Medical Sciences, College of Medicine National Cheng Kung University Tainan Taiwan; ^5^ Center of Transformative Bioelectronic Medicine National Cheng Kung University Tainan Taiwan; ^6^ Department of Neurosurgery Chiayi Christian Hospital Chiayi Taiwan; ^7^ Department of General Psychiatry, Jianan Psychiatric Center Ministry of Health and Welfare Tainan Taiwan; ^8^ Department of Psychiatry, National Cheng Kung University Hospital, College of Medicine National Cheng Kung University Tainan Taiwan; ^9^ Department of Neurosurgery Tainan Municipal Hospital (Managed by Show Chwan Medical Care Corporation) Tainan Taiwan

**Keywords:** depression, memory deficit, sciatic nerve stimulation, stress

## Abstract

Stress causes depression and cognitive decline. With limitations in pharmacotherapy, sciatic nerve stimulation (SNS) offers a promising nondrug alternative. This study aimed to explore the therapeutic efficacy of SNS in mitigating stress‐induced depressive behaviors and memory deficits by focusing on astrocytic dysfunction and cellular senescence in the hippocampus. C57BL/6 mice were subjected to the water immersion restraint stress (WIRS) paradigm to induce stress‐related behavioral deficits. Behavioral tests assessed locomotion, anxiety, depression‐like behavior, and memory. Astrocytic disruption and cellular senescence in the hippocampus were assessed using glial fibrillary acidic protein (GFAP) immunostaining and senescence‐associated β‐galactosidase (SA‐β‐gal) staining. SNS at 20 Hz significantly improved cognitive function and reduced depression‐like behavior in WIRS‐treated mice. It also restored hippocampal GFAP expression and decreased both SA‐β‐gal‐positive cell accumulation and the expression of senescence markers p16 and p21, suggesting an attenuation of cellular senescence. To further explore the link between cellular senescence and SNS‐mediated effects, we administered the anti‐senescence agent vitamin C to WIRS mice. While vitamin C alleviated stress‐induced hippocampal senescence and depressive behavior, it failed to reverse memory deficits or restore GFAP expression, indicating that the benefits of SNS extend beyond its anti‐senescent actions. In summary, SNS effectively counteracts the neurobehavioral consequences of chronic stress by targeting astrocytic dysfunction and cellular senescence. These findings support SNS as a promising, nonpharmacological strategy for treating stress‐related depression and cognitive decline. Future studies should explore its clinical translation and broader therapeutic potential.

**Abbreviations**
CA: cornu ammonis
DBS: deep brain stimulation
DG: dentate gyrus
ECT: electroconvulsive therapy
FST: forced swimming test
GFAP: glial fibrillary acidic protein
OLRT: object location recognition test
PBST: phosphate‐buffered saline containing 0.3% Triton X‐100
PSD: post‐SNS day
rTMS: repetitive transcranial magnetic stimulation
SA‐β‐gal: senescence‐associated β‐galactosidase
SD: standard deviations
SNS: sciatic nerve stimulation
TST: tail suspension test
WIRS: water immersion restraint stress

## 1 Introduction

Stress impacts health through autonomic and neuroendocrine pathways and alters health behaviors, including depression and memory decline. Evidence shows that neuroplasticity, crucial for neuronal adaptation, is disrupted in both mood disorders and stress models [1]. The role of stress or dysregulated stress responses in driving molecular, cellular, and behavioral alterations linked to depression has become increasingly evident. Memory decline has also been linked to the stress‐induced disruption of neuroplasticity [2]. Pharmacological treatments targeting maladaptive neuroplasticity have been developed for depression and memory decline, yet they face significant clinical challenges. First‐line antidepressants, which boost neuroplasticity by increasing monoamine availability [3], often suffer from delayed effects, low efficacy, poor adherence [4], and severe side effects [5]. Consequently, many studies have focused on exploring alternative nonpharmacological strategies.

Electrical neural stimulation is an effective method for promoting neuroplasticity both in vitro and in vivo [6], such as electroconvulsive therapy, repetitive transcranial magnetic stimulation (rTMS), deep brain stimulation (DBS), and vagus nerve stimulation have been suggested or used to treat drug‐resistant depression [7] and dementia [8]. However, these nonpharmacological therapies often yield inconsistent treatment outcomes and can cause nonnegligible side effects. For instance, rTMS has been associated with an elevated risk of seizure induction [9], whereas DBS has been linked to an increased risk of anxiety, insomnia, and cognitive decline [10]. These shortcomings underscore the urgent need for safer neural stimulation approaches for treating depression and cognitive decline. Notably, our previous study found that neuromodulation using sciatic nerve stimulation (SNS) at 20 Hz activates serotonergic neurons in the raphe magnus nucleus [6] through an ascending pathway from the peripheral nerves to the brainstem and other central structures [11]. Increasing synaptic serotonin availability is essential for ameliorating depressive symptoms and cognitive impairments in neuropsychiatric disorders [12]. These findings suggest that the SNS may be a promising treatment strategy for stress‐related depression and memory decline, although the therapeutic effects and underlying mechanisms of action of SNS in these conditions remain unclear.

Astrocytes are highly susceptible to chronic stress [13] and astrocytic dysfunction plays a crucial role in the pathogenesis of depression and cognitive impairment. Studies have reported that psychosocial stress can reduce the number of glial fibrillary acidic protein (GFAP)‐positive astrocytes in the hippocampus of tree shrews [14]. Chronic unpredictable stress reduced GFAP‐expressing astrocyte density in the prefrontal cortex of rats, inducing depression‐like behavior. These findings align with those of postmortem studies showing a significant reduction in the number of glial cells in the prefrontal cortex of individuals with a history of depression [15]. Collectively, a reduction in GFAP‐positive astrocytes may drive stress‐related behavioral changes and contribute to the development of mood disorders under certain conditions.

Cellular senescence is proposed as a factor in mental illness and memory impairment [16]. This process involves cell cycle arrest accompanied by distinct morphological, cellular, and molecular changes [17]. Unlike other nondividing cells, senescent cells exhibit unique markers and morphological features, including senescence‐associated β‐galactosidase (SA‐β‐gal) activity. Interestingly, chronic stress has been implicated as a trigger for cellular senescence [18]. Studies showed that chronic unpredictable stress induces depression‐like behaviors and memory deficits in mice, along with the accumulation of senescent cells. Memory impairment was strongly correlated with the number of senescent hippocampal cells, and clearance of these cells resulted in the reversal of stress‐induced memory decline [19]. These findings suggest that cellular senescence may present a potential therapeutic target.

In this study, we aimed to investigate SNS's potential to alleviate stress‐induced depression and memory impairment. We examined the astrocytic dysfunction and cellular senescence in the hippocampus, a brain region critical for both depression‐like behavior [20] and cognitive processes such as spatial learning and memory [21]. We employed the water immersion restraint stress (WIRS) paradigm to induce stress‐related deficits and utilized various behavioral tests, including the open‐field test (OFT), forced swimming test (FST), tail suspension test (TST), Y‐maze, and object location recognition test (OLRT), to evaluate locomotor activity, anxiety, depression‐like behavior, and memory function. Astrocytic disruption in the mouse hippocampi was evaluated using GFAP immunostaining, while cellular senescence was assessed through SA‐β‐gal staining and immunoblotting for p16 and p21 [22]. To further investigate the role of senescence, a cohort of WIRS mice was treated with the anti‐senescence agent vitamin C [23]. This study provides insights for future neural stimulation interventions to mitigate stress‐induced depression and cognitive decline.

## 2 Materials and Methods

### 2.1 Animals

Animal experiments were approved by the IACUC of National Cheng Kung University (113012). Eight‐week‐old male C57BL/6N mice were housed (5/cage) in an AAALAC‐accredited facility under controlled conditions (55% ± 10% humidity, 24°C ± 1°C, 13‐h light/11‐h dark cycle) with ad libitum food and water. A two‐factorial design [WIRS vs. CTRL × SNS vs. Sham] was used, with mice randomly assigned to four groups: CTRL‐Sham, CTRL‐SNS, WIRS‐Sham, and WIRS‐SNS. An additional cohort of WIRS mice was treated with the anti‐senescence agent vitamin C to further investigate the role of cellular senescence. Randomization was performed using Microsoft Excel.

### 2.2 Water Immersion Restraint Stress

A 14‐day WIRS protocol induced depression‐like behavior and memory deficits. WIRS mice were immobilized in ventilated 50‐mL tubes and submerged (0.5 cm) for 8 h daily, while CTRL mice remained in home cages under identical conditions. A pilot study confirmed WIRS efficacy, showing increased immobility in FST and TST (Figure S1).

### 2.3 Sciatic Nerve Electrical Stimulation (SNS)

One day after the conclusion of the WIRS procedure, the mice underwent SNS treatment. They were anesthetized with tiletamine‐zolazepam (Zoletil, 50 mg/kg, i.p.; Virbac, Carros, Alpes‐Maritimes, France) and xylazine (2.5 mg/kg, i.p.; Bayer AG, Leverkusen, Nordrhein‐Westfalen, Germany). An incision was made on the right thigh of each mouse to expose the sciatic nerve, which was then hooked to a customized bipolar electrode connected to an electromyography stimulator (Model#: ISIS Xpress, inomed Medizintechnik GmbH, Emmendingen, Baden‐Württemberg, Germany). Stimulation was delivered as uniform, 2‐s biphasic pulse trains at a frequency of 20 Hz, with 200‐μs square wave pulses, followed by 8‐s pauses. This stimulation protocol lasted for 30 min, with voltages ranging from 0.5 to 2 V and current intensities ranging between 1 and 10 mA (Figure S2). Successful stimulation was confirmed by motor responses, and the intensity was set at the highest level that did not induce a motor response. These parameters were adapted from previous studies that showed activation of all fiber types. After electrical stimulation, the incision was closed with 4–0 silk sutures. In the Sham mice, electrodes were placed over the sciatic nerve without delivering any electrical current.

### 2.4 Vitamin C Treatment

One day following the completion of the WIRS procedure, a cohort of mice received vitamin C treatment as a positive control for anti‐senescence effects. Vitamin C (Cat#: A7506, Sigma‐Aldrich, St. Louis, MO, USA), prepared in PBS, was administered via a single intraperitoneal injection at a dose of 100 mg/kg.

### 2.5 Open‐Field Test

On post‐SNS day (PSD) 1, OFT assessed spontaneous locomotion. Mice explored a 46 × 46 × 46 cm white box for 10 min. Total distance, central zone distance/time (25% area), and entry latency were analyzed using Noldus EthoVision XT.

### 2.6 Object Location Recognition Test

From PSD 1 to 3, OLRT assessed hippocampus‐associated long‐term spatial memory. On Day 1, mice acclimated in a 30 × 30 × 30 cm box for 10 min. On Day 2, they explored two identical objects on the same side for 10 min. After 24 h, one object was relocated, and mice explored for another 10 min. The box was cleaned with 70% ethanol between trials. The time spent exploring each object was recorded, and the discrimination index was calculated as follows: (exploration time_novel location_ − exploration time_familiar location_)/(total exploration time).

### 2.7 Y‐Maze Test

On PSD 3, hippocampus‐associated short‐term spatial reference memory was assessed using a Y‐maze. Mice first explored two open arms for 5 min while one arm was blocked. After 1 h, the blocked arm was opened, and mice explored all three arms for 5 min. The time spent in the newly opened arm within the first 2 min was recorded as a percentage of total exploration time.

### 2.8 Forced Swimming Test

On PSD 4, FST assessed behavioral despair. Mice were placed in 22°C water (30 cm × 20 cm) for 10 min, and immobility duration and percentage were analyzed using the SMART tracking system.

### 2.9 Tail Suspension Test

On PSD 4, TST was performed as described previously. Mice were suspended 60 cm above the floor for 6 min using adhesive tape near the tail tip. Behavior was recorded, and immobility duration and percentage were analyzed with the SMART tracking system.

### 2.10 Sample Collection

On PSD 5, mice were anesthetized with tiletamine‐zolazepam (70 mg/kg) and xylazine (2.5 mg/kg), then perfused transcardially with ice‐cold saline. Brains were then collected and bisected into hemispheres. One hemisphere was rapidly frozen in liquid nitrogen and stored at −80°C for subsequent Western blot analysis. The other hemisphere was fixed in 4% paraformaldehyde in phosphate buffer at 4°C for 48 h. Following fixation, tissues were dehydrated in sucrose solutions and sectioned into 20‐μm slices using a cryostat (Model#: CM1950, Leica Biosystems, Nussloch, Baden‐Württemberg, Germany).

### 2.11 Immunofluorescence Staining

Brain sections were rinsed with PBST and pretreated with saline‐sodium citrate buffer at 85°C for 15 min. The sections were first incubated in PBST with 3% normal goat serum for 1 h, followed by overnight incubation with primary antibodies against GFAP (1:400 dilution prepared in PBST, Cat#: G9269, Sigma‐Aldrich) at room temperature. On the following day, the sections were hybridized with Alexa Fluor 594‐conjugated goat anti‐rabbit IgG (1:200 dilution prepared in PBST, Cat#: 111‐585‐003, Jackson ImmunoResearch Laboratories, West Grove, PA, USA) for 2 h at room temperature, washed with PBST, and mounted in media containing 4′,6‐diamidino‐2‐phenylindole (DAPI, Cat#: ab104139, Abcam, Cambridge, UK). GFAP‐immunoreactive area fractions within regions of interest were quantified using ImageJ software (v2.0.0‐rc‐69/1.52p, U.S. National Institutes of Health, Bethesda, MD, USA).

### 2.12 Senescence‐Associated β‐Galactosidase Activity Assay

Brain sections were rinsed with PBS and incubated overnight in SA‐β‐gal staining solution (Cat#: 9860, Cell Signaling Technology, Danvers, MA, USA) at 37°C. After staining, the SA‐β‐gal solution was discarded, and the sections were mounted with 70% glycerol and stored at 4°C. SA‐β‐gal‐positive area fractions within regions of interest were quantified using ImageJ software (v2.0.0‐rc‐69/1.52p, U.S. National Institutes of Health).

### 2.13 Nissil Staining

Brain sections were mounted on coated slides and incubated overnight in a 1:1 alcohol/chloroform solution to minimize background staining. The sections were then rehydrated through a graded ethanol series (100% and 95%) followed by distilled water and subsequently stained with 0.1% cresyl violet solution (Cat#: ab246816, Abcam) in a 50°C oven for 10 min. After staining, sections were briefly rinsed with distilled water, dehydrated from 100% alcohol to xylene, and coverslipped using a permanent mounting medium (Cat#: 3801101, Leica Biosystems).

### 2.14 Western Blots

Frozen hippocampal tissues were homogenized in ice‐cold T‐PER lysis buffer (Cat#: 78510, Thermo Fisher Scientific Inc., Waltham, MA, USA) supplemented with protease inhibitors (Cat#: 04693116001, Roche, Basel, Switzerland) and phosphatase inhibitors (Cat#: PHOSS‐RO, Roche). The homogenates were centrifuged at 14,000 × *g* for 10 min at 4°C, and the resulting supernatants were collected. Protein concentrations were determined using a commercial protein assay kit (Cat#: 23225, Thermo Fisher Scientific Inc.). For each sample, 30 μg of total protein was mixed with sample buffer (Cat#: S3401, Sigma‐Aldrich), denatured by boiling, and loaded onto a 12% polyacrylamide gel. Proteins were separated by electrophoresis for 2 h and transferred onto PVDF membranes (Cat#: IPVH00010, Merck‐Millipore, Burlington, MA, USA) using the wet transfer method. Membranes were incubated overnight at 4°C with primary antibodies against p16 (Cat#: 23200, Cell Signaling Technology, Danvers, MA, USA), p21 (Cat#: 64016, Cell Signaling Technology), and α‐tubulin (Cat#: GTX112141, GeneTex, Irvine, CA, USA). Densitometric analysis was performed using ImageJ software (v2.0.0‐rc‐69/1.52p, U.S. National Institutes of Health).

### 2.15 Statistical Analysis

All data are presented as mean ± standard deviation (SD). Statistical analyses and graph plotting were conducted using the Prism software (v. 10, GraphPad Software Inc., San Diego, CA, USA). Statistical significance was set at *p* < 0.05. An ordinary two‐way ANOVA followed by Tukey's multiple comparison test was employed to analyze all datasets with two independent factors. An ordinary one‐way ANOVA followed by Tukey's multiple comparison test was employed to analyze the datasets with more than two groups. The sample sizes are indicated in the accompanying scatter plots.

## 3 Results

### 3.1 Neither WIRS nor SNS Affects Anxiety Levels or Locomotor Activity in mice

**FIGURE 1:**
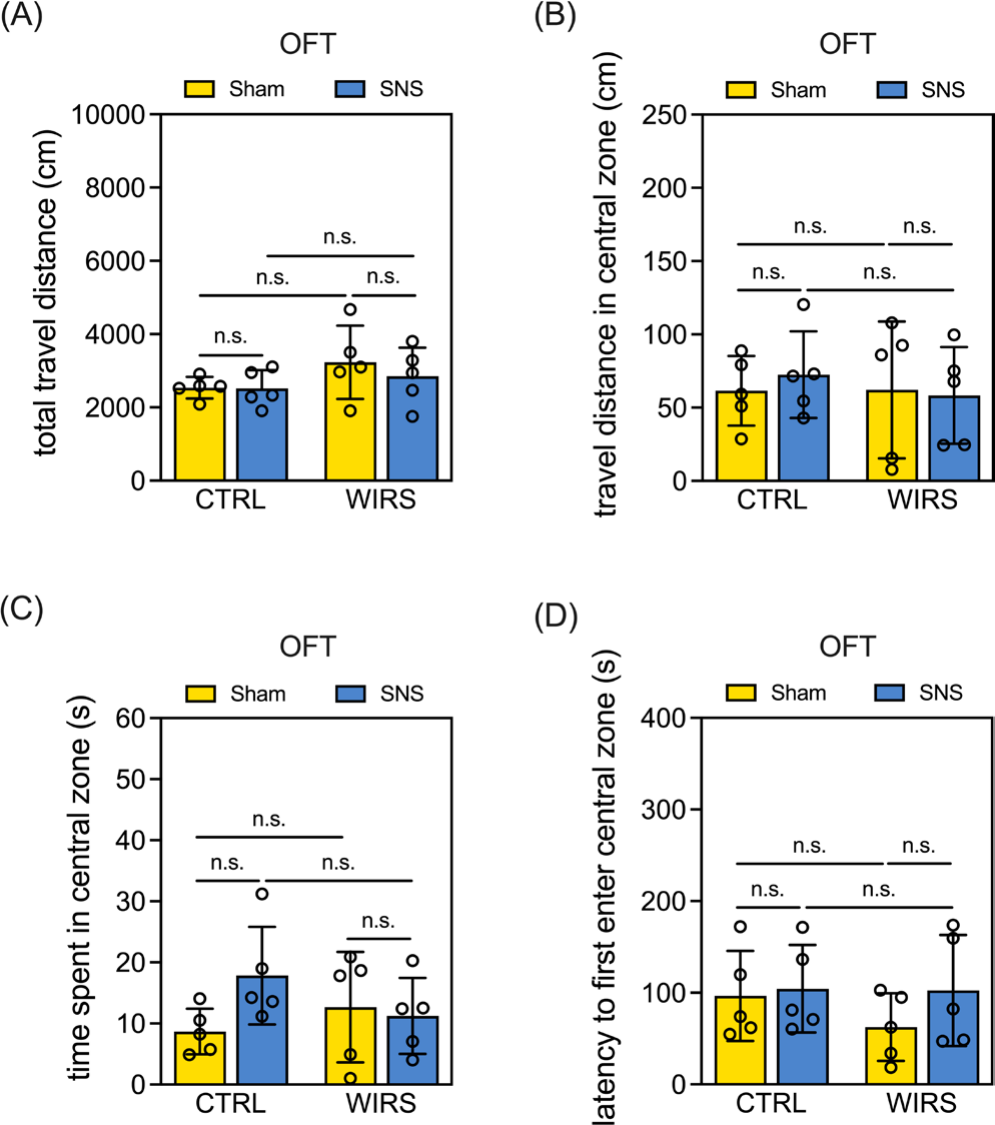
Neither WIRS nor SNS affects anxiety levels or locomotor activity in mice. (A) Quantitative results of the total travel distance in the OFT. (B) Quantitative results of the travel distance in the central zone in the OFT. (C) Quantitative results of time spent in the central zone in the OFT. (D) Quantitative results of latency to first enter the central zone in the OFT. Data are expressed as the mean ± SD. Sample size = 5 mice per group; n.s.: not significant.

Chronic stress is known to induce anxiety symptoms, and SNS surgery for the hind limbs of mice could potentially influence locomotor activity, complicating the interpretation of behavioral tests. To eliminate this possibility, we conducted the OFT to assess anxiety‐like behaviors and ensure that SNS did not impair locomotor function. Our findings revealed that neither WIRS nor SNS affected the total distance traveled (Figure 1A), distance traveled in the central zone (Figure 1B), time spent in the central zone (Figure 1C), or the latency to first enter the central zone (Figure 1D). These results suggest that neither WIRS nor SNS affected anxiety levels or locomotor activity in mice.

### 3.2 SNS Alleviates WIRS‐Induced Exhibition of Depression‐Like Behaviors and Spatial Memory Impairments in Mice

**FIGURE 2:**
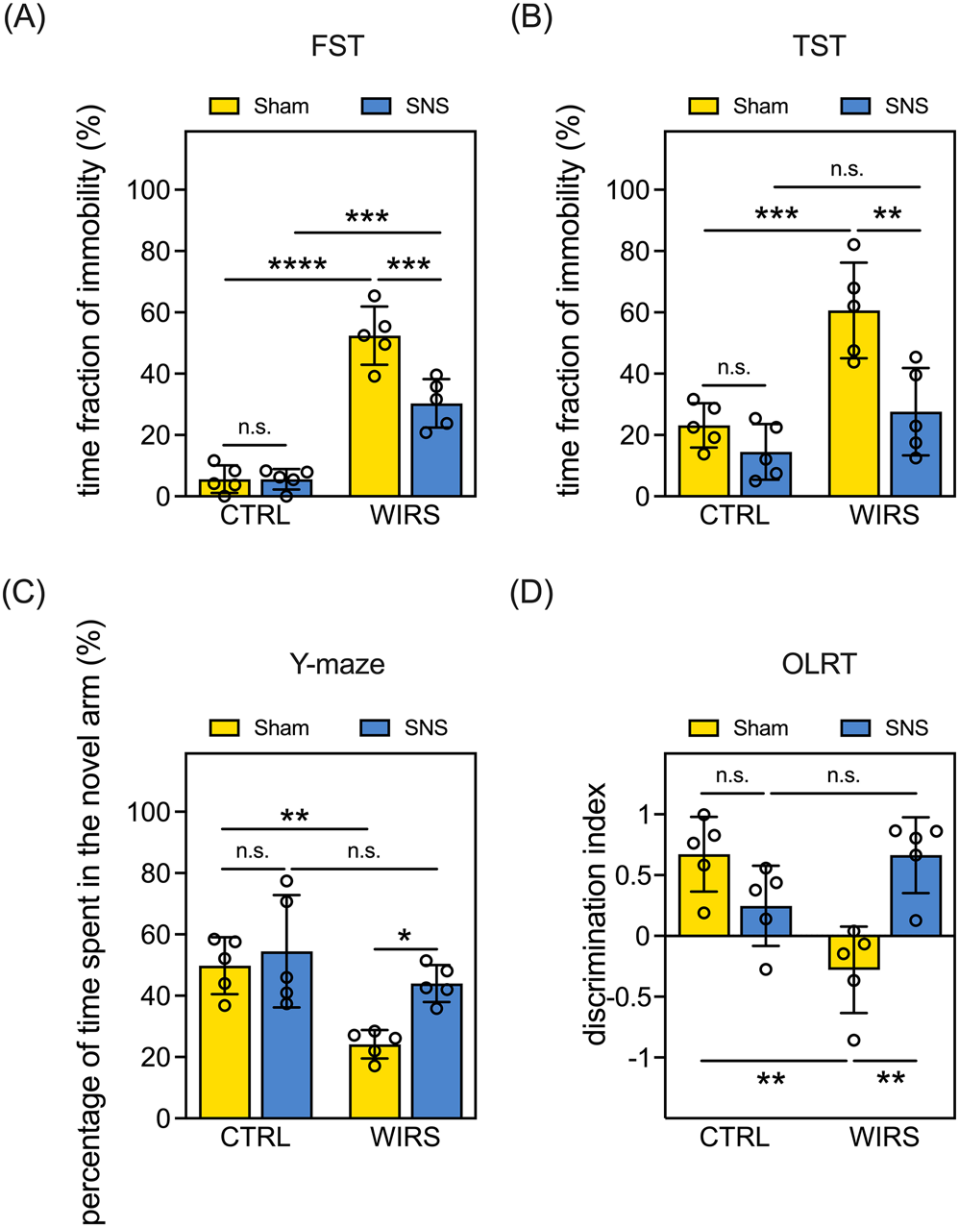
SNS alleviates WIRS‐induced exhibition of depression‐like behavior and spatial memory impairment in mice. (A) Quantitative results of the time fraction of immobility in the FST. (B) Quantitative results of the time fraction of immobility in the TST. (C) Quantitative results of the percentage of time spent in the novel arm in the Y‐maze test. (D) Quantitative results of the discrimination index in the OLRT. Data are expressed as the mean ± SD. *p < 0.05, **p < 0.01, ***p < 0.001, ****p < 0.0001. Sample size = 5 mice per group; n.s.: not significant.

The interactive effects of WIRS and SNS on the exhibition of depression‐like behaviors were examined using the FST and TST. The WIRS‐induced behavioral despair manifested as an increase in the time fraction of immobility in the FST and TST (WIRS‐Sham vs. CTRL‐Sham), which was reversed by SNS (WIRS‐SNS vs. WIRS‐Sham; Figure 2A,B). SNS did not affect FST or TST performance in unstressed CTRL mice (CTRL‐SNS vs. CTRL‐Sham; Figure 2A,B).

The Y‐maze test and OLRT were used to assess hippocampus‐related spatial memory. In the Y‐maze test, WIRS‐Sham mice spent less time in the novel arm than CTRL‐Sham mice (Figure 2C), a deficit that was reversed by SNS treatment (WIRS‐SNS vs. WIRS‐Sham, Figure 2C). Similarly, in the OLRT, WIRS reduced the time spent exploring the object in the novel location, as indicated by the lower discrimination index (WIRS‐Sham vs. CTRL‐Sham, Figure 2D). This effect was also reversed by SNS treatment (WIRS‐SNS vs. WIRS‐Sham; Figure 2D). SNS alone did not affect the performance of unstressed CTRL mice in either the Y‐maze test or the OLRT (CTRL‐SNS vs. CTRL‐Sham; Figure 2C,D). Collectively, we suggest that SNS alleviates WIRS‐induced depression‐like behavior and spatial memory impairment in mice.

### 3.3 SNS Restores WIRS‐Induced Reduction in GFAP Expression in the Hippocampi of Mice

**FIGURE 3:**
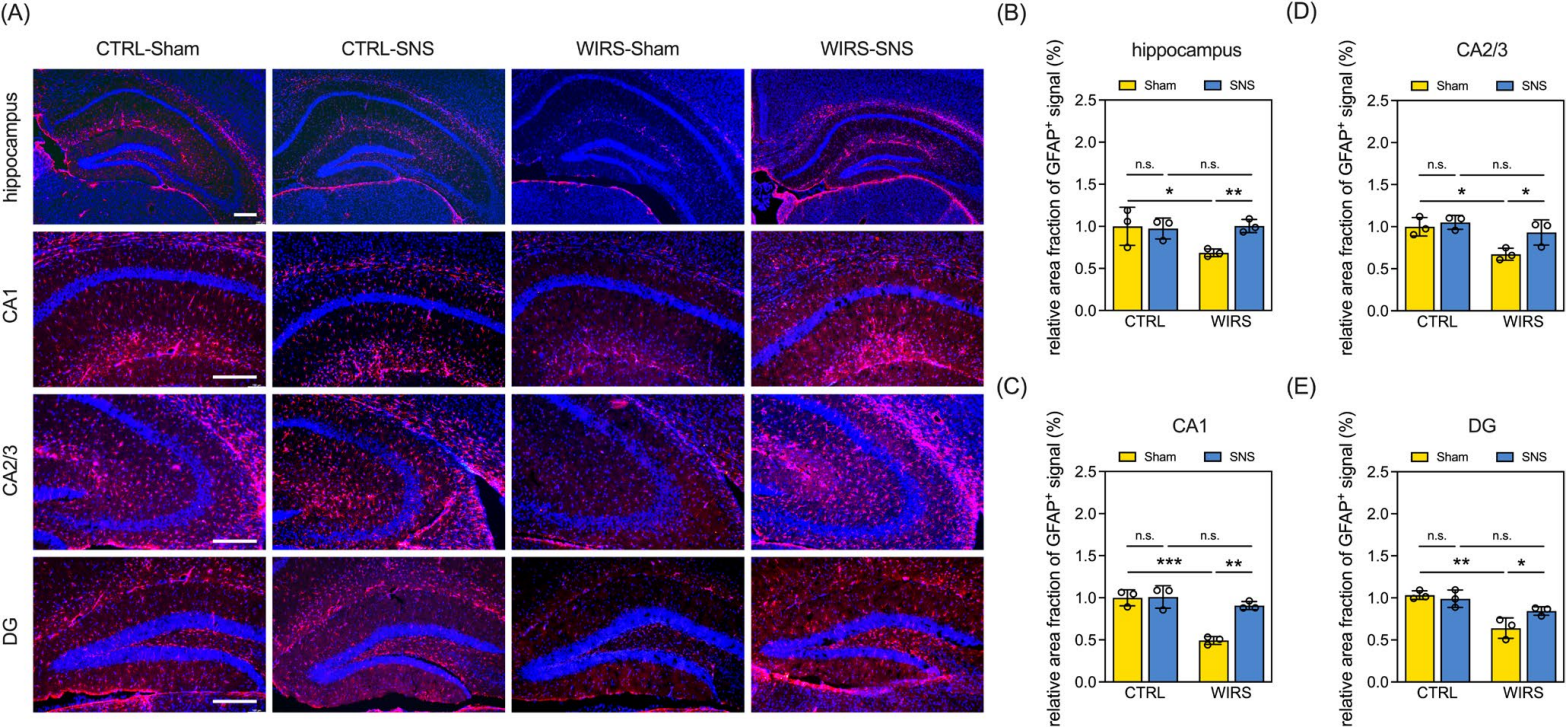
SNS restores WIRS‐induced reduction in GFAP expression in the hippocampi of mice. (A) Representative micrographs of immunofluorescence staining for GFAP (red) in the hippocampus and its subregions with DAPI (blue). Scale bar = 250 μm. (B–E) Quantitative results of the relative area fraction of GFAP^+^ signaling in the (B) whole hippocampus, (C) CA1, (D) CA2/3, and (E) DG. Data are expressed as the mean ± SD. *p < 0.05, **p < 0.01, ***p < 0.001 Sample size = 3 mice per group; n.s.: not significant.

Based on our behavioral findings, we hypothesized that alterations in the hippocampus, a region essential for regulating depressive behaviors and spatial memory, may underlie the deficits induced by WIRS and the therapeutic effects of SNS. To test this, we assessed hippocampal expression of GFAP, an astrocytic marker known to be associated with the pathophysiology of depression and cognitive dysfunction [24, 25, 26]. We found that WIRS downregulated GFAP expression in the hippocampi of mice (WIRS‐Sham vs. CTRL‐Sham, Figure 3A,B). Specifically, this WIRS‐induced decrease in GFAP expression was evident across various hippocampal subregions, including the cornu ammonis (CA) 1, CA2/3, and dentate gyrus (DG) (WIRS‐Sham vs. CTRL‐Sham, Figure 3A,C–E). Importantly, SNS treatment restored GFAP expression levels in the entire hippocampus and the three selected subregions in WIRS mice (WIRS‐SNS vs. WIRS‐Sham, Figure 3A–E). Consistent with the behavioral tests, the level of GFAP in the hippocampus was not affected by SNS in the CTRL mice (CTRL‐SNS vs. CTRL‐Sham, Figure 3A–E). These results suggest that SNS restores WIRS‐induced reduction in GFAP expression in the mouse hippocampus.

### 3.4 SNS Reverses WIRS‐Induced Cellular Senescence in the Hippocampi of Mice

**FIGURE 4:**
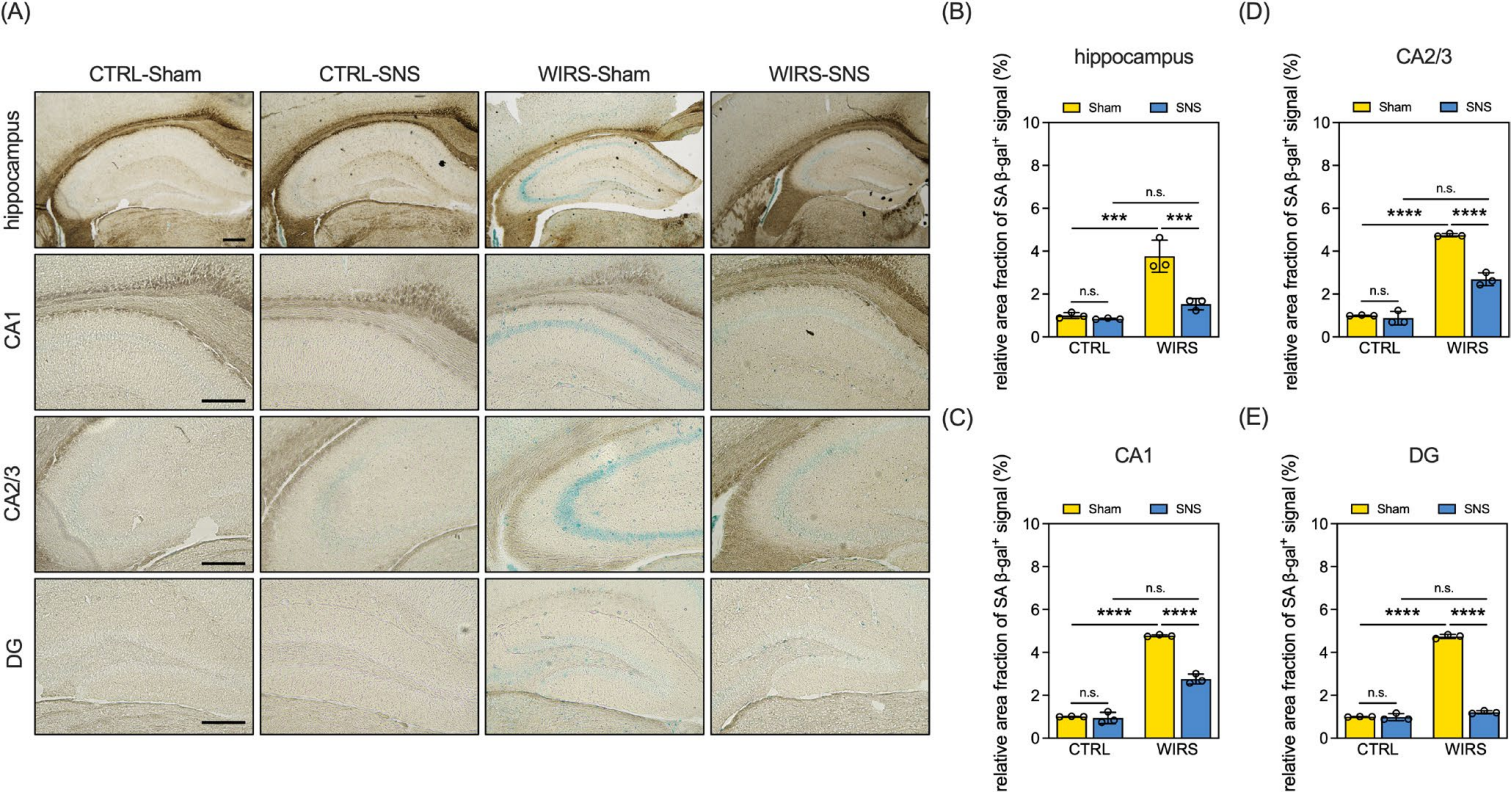
SNS reversed WIRS‐induced cellular senescence in the hippocampi of mice (A) Representative micrographs of SA‐β‐gal staining in the hippocampus and its subregions. Scale bar = 250 μm; (B‐E) Quantitative results of relative area fraction of SA‐β‐gal^+^ signaling in the (B) whole hippocampus, (C) CA1, (D) CA2/3, and (E) DG. Data are expressed as mean ± SD. ***p < 0.001, ****p < 0.0001; Sample size = 3 mice per group; n.s.: not significant.

Next, we explored the effects of WIRS and SNS on cellular senescence in the hippocampus, as the presence of SA‐β‐gal‐positive signals in this region is implicated in the development of depressive phenotypes and memory decline. We found that WIRS increased the area fraction of SA‐β‐gal‐positive signals in the hippocampi of mice (WIRS‐Sham vs. CTRL‐Sham, Figure 4A,B). This WIRS‐induced increase in SA‐β‐gal‐positive signals was identified in the hippocampal subregions, including CA1, CA2/3, and DG (WIRS‐Sham vs. CTRL‐Sham, Figure 4A,C–E). Importantly, SNS treatment reduced SA‐β‐gal‐positive signals in the entire hippocampus and these specific subregions in WIRS mice (WIRS‐SNS vs. WIRS‐Sham, Figure 4A–E). Consistent with the patterns of behavioral performance and GFAP expression, SNS did not affect cellular senescence levels in the hippocampus of unstressed CTRL mice (CTRL‐SNS vs. CTRL‐Sham, Figure 4A–E). Furthermore, Nissl staining confirmed that neither WIRS nor SNS resulted in overt neuronal loss in the hippocampus (Figure S3). These results suggest that SNS reversed WIRS‐induced cellular senescence in the mouse hippocampus.

### 3.5 Vitamin C Attenuates Hippocampal Cellular Senescence and Depressive‐Like Behavior but Fails to Restore Memory Function or GFAP Expression in WIRS Mice

To further explore the relationship between hippocampal cellular senescence and other stress‐induced alterations, we included an additional cohort of mice, designated as the WIRS‐C group. These mice received a single intraperitoneal injection of the anti‐senescence agent vitamin C (100 mg/kg) 1 day after the conclusion of the WIRS procedure and subsequently underwent the same behavioral and histological assessments as the other experimental groups.

Our findings revealed that vitamin C treatment significantly reduced WIRS‐induced cellular senescence in the hippocampus, particularly within the CA1 and CA2/3 subregions, as evidenced by decreased SA‐β‐gal staining (WIRS‐C vs. WIRS‐Sham, Figure S4A–D). However, this anti‐senescence effect was less pronounced in the dentate gyrus (DG), where no significant improvement was observed (WIRS‐C vs. WIRS‐Sham, Figure S4A,E). Notably, SNS intervention exhibited a stronger suppressive effect on SA‐β‐gal‐positive signals compared to vitamin C treatment (WIRS‐SNS vs. WIRS‐C, Figure S4A–E). We further assessed the expression of two key senescence markers, p16 and p21, in the hippocampus using Western blot analysis. WIRS markedly increased the levels of both markers relative to controls (WIRS‐Sham vs. CTRL‐Sham, Figure S4F–H), and these elevations were significantly reversed by SNS treatment (WIRS‐SNS vs. WIRS‐Sham, Figure S4F–H). Vitamin C also attenuated the upregulation of p16 and p21 (WIRS‐C vs. WIRS‐Sham, Figure S4F,I,J), though its effect was less robust than that of SNS (WIRS‐C vs. WIRS‐SNS, Supplementary Figure S4F,I,J), supporting its use as a reference anti‐senescence control in our study.

Using the WIRS‐C group, we further examined whether suppression of cellular senescence was sufficient to restore other stress‐induced impairments. While neither WIRS, SNS, nor vitamin C affected locomotor activity in the open field test (Figure 1 and Figure S5A–D), vitamin C significantly reversed WIRS‐induced behavioral despair in the FST and TST (WIRS‐C vs. WIRS‐Sham, Figure S5E,F). However, it failed to rescue cognitive deficits observed in the Y‐maze and OLRT (WIRS‐C vs. WIRS‐Sham, Figure S5G,H). Most critically, unlike SNS, vitamin C treatment did not restore the hippocampal level of GFAP (WIRS‐C vs. WIRS‐Sham, Figure S6).

Collectively, these results indicate that while attenuation of hippocampal cellular senescence via vitamin C is sufficient to alleviate depression‐like behavior, it does not restore memory function or astrocytic integrity as reflected by GFAP expression. This suggests a dissociation between the reversal of cellular senescence and the recovery of other stress‐related phenotypes. The inclusion of the WIRS‐C group thus helped reveal the complexity of the mechanistic landscape, highlighting that the restoration of astrocytic function and cognitive performance may require broader or distinct cellular targets beyond the suppression of senescence alone.

## 4 Discussion

We utilized a chronic WIRS paradigm to induce depression‐like behaviors and impairments in hippocampus‐related short and long memory in mice. We demonstrated that a single cycle of 20‐Hz SNS effectively corrected these WIRS‐induced behavioral deficits. Moreover, we found that SNS reversed WIRS‐induced GFAP loss and cellular senescence, which are major pathological hallmarks associated with depression and memory decline in the hippocampus of mice. Collectively, our results indicate that not only does SNS exert positive influences on behavioral outcomes, but it also confers neuroprotective benefits.

Studies consistently show depression is strongly linked to hippocampal dysregulation [27]. Stress, elevated glucocorticoids, changes in neurotrophic factors and glial transporters, and neurotransmitter fluctuations alter glial populations and neurophysiology in depression [28]. We observed significantly reduced GFAP levels in the DG, CA1, and CA2/3 regions of the hippocampus in the WIRS model. Consistent with our results, previous studies show that chronic stress reduces GFAP mRNA expression in the prefrontal cortex [29] and GFAP protein levels in the hippocampus [30]. Chronic stress also induces morphological changes in GFAP‐immunoreactive astrocytes in wild‐type mice [31]. Diminished glial cell density and number have been observed in the frontolimbic regions of human patients with major depression. Additionally, younger individuals with depression exhibit age‐dependent reductions in GFAP density and lower GFAP levels in the prefrontal cortex [28]. GFAP levels were seven times lower in younger (aged < 60 years) individuals with major depressive disorder than those in older patients and age‐matched controls [32]. Reduced GFAP staining has also been reported in the hippocampi of patients with depression [33], particularly in the DG of women with major depressive disorder, with a similar trend observed in the CA2/3 region [34]. The reduction in the astrocyte population can impair glutamate uptake, potentially leading to excitotoxic neuronal damage [35]. Furthermore, glucocorticoid receptor‐α protein is co‐localized in approximately 20% of astrocytes in the DG and 50% in the CA subfields, suggesting that astrocytes may be particularly susceptible to elevated glucocorticoid levels and become dysfunctional following chronic stress [36]. Notably, we show that SNS significantly restored GFAP expression in the hippocampus of WIRS mice but did not alter GFAP levels in the hippocampus of nonstressed naive mice. Ethridge et al. found that a single 30‐min session of 0.25‐mA transcranial direct current stimulation did not alter GFAP immunoreactivity in the hippocampi of rats [37]. These results, along with ours, suggest that SNS restores hippocampal GFAP levels by targeting the same pathway responsible for WIRS‐induced GFAP loss. Moreover, both SNS and transcranial direct current stimulation appear to have no effect on astrocytic function in healthy animals, supporting the safety of these neural stimulation therapies. To further advance our understanding, it is crucial to explore the shared molecular pathways involved in both WIRS‐induced GFAP loss and SNS‐induced restoration of GFAP expression, as they may reveal key therapeutic targets for depression and stress‐related disorders.

Stress triggers cellular senescence, closely linking depressive disorders and cognitive decline. We found a significant increase in SA‐β‐gal‐positive cells in the CA3 and DG regions of WIRS‐treated mice compared to CTRL mice. SNS treatment significantly reduced WIRS‐induced senescence in the hippocampus. Previous studies show that prolonged stress, especially in aging, drives neurons, astrocytes, and microglia into chronic cellular senescence. Studies have also identified a high number of SA‐β‐gal‐positive cells in the DG, particularly in the hilus, of mice exposed to chronic stress [38]. Similarly, the chronic unpredictable mild stress model showed elevated cellular senescence and γ‐H2AX activity in the hippocampus compared to CTRL mice [39]. In the MRL/lpr mouse model of systemic lupus erythematosus, which also exhibits depressive behavior, senescent neural cells accumulated in the CA3 region [40]. Targeting and removing senescent cells is a promising therapeutic strategy to counter aging, prevent age‐related diseases, and improve cognitive function in mouse models [19]. Collectively, our findings highlight the critical role of stress‐induced cellular senescence in the hippocampus, particularly in relation to depressive behavior and cognitive decline. The ability of SNS to mitigate the WIRS‐induced senescent response suggests its potential as a therapeutic intervention.

Despite the observed efficacy of vitamin C in attenuating hippocampal cellular senescence and reversing depressive‐like behaviors in WIRS mice, it failed to restore cognitive performance or hippocampal GFAP expression. These findings highlight important limitations in targeting senescence alone as a therapeutic strategy. While the antidepressant‐like effects of vitamin C suggest a potential GFAP‐independent mechanism, its inability to ameliorate cognitive deficits raises the possibility that astrocytic dysfunction, particularly the loss of GFAP expression, may play a causal role in stress‐induced memory impairment. Moreover, the dissociation between senescence attenuation and astrocytic restoration uncovered by the WIRS‐C group underscores the complexity of the cellular and molecular interactions underlying stress‐related neuropathology. These results suggest that reducing cellular senescence is necessary but not sufficient to fully reverse the spectrum of WIRS‐induced deficits. Further investigation is warranted to delineate the distinct and potentially interdependent contributions of astrocytic integrity and cellular senescence to mood and cognitive outcomes, which may inform the development of more comprehensive therapeutic interventions.

We found that SNS of 20 Hz effectively mitigated depression‐like behaviors and memory deficits. This intervention not only improves cognitive and emotional outcomes, but also restores GFAP expression and reduces cellular senescence in the hippocampus, highlighting its multifaceted nature, which targets both the symptoms and underlying neuropathology associated with these debilitating conditions. These findings underscore the potential of SNS as an innovative therapeutic approach for treating depression and cognitive impairment.

## Conflicts of Interest

The authors declare no conflicts of interest.

## Supporting information


**Figure S1:** Water Immersion Restraint Stress induced depression‐like behavior in a rodent model. Representative (A) WIRS‐treated mice showed significant immobility compared with nonstressed control mice, as suggested by the Forced Swimming Test (control groups: *n* = 9; WIRS groups: *n* = 6). (B) WIRS‐treated mice showed significant immobility compared with nonstressed control mice, as suggested by the Tail Suspension Test (control groups: *n* = 8; WIRS groups: *n* = 5). Data are expressed as mean ± SD. *p < 0.05, **p < 0.01, ***p < 0.001.

**Figure S2:** Parameters of the SNS waveform.

**Figure S3:** Neither WIRS nor SNS lead to an obvious neuronal loss in the hippocampus of mice. The representative Nissl staining images. Scale: 250 μm.

**Figure S4:** Vitamin C attenuates hippocampal cellular senescence in WIRS mice. (A) Representative micrographs of SA‐β‐gal staining in the hippocampus and its subregions. Scale bar = 250 μm; (B–E) Quantitative results of relative area fraction of SA‐β‐gal^+^ signaling in the (B) whole hippocampus, (C) CA1, (D) CA2/3, and (E) DG. (F) Representative Western blot images showing hippocampal expression of senescence markers p16 and p21. (G, H) Quantification of the effects of WIRS and SNS on hippocampal levels of (G) p16 and (H) p21. (I, J) Quantification of the effects of WIRS, SNS, and vitamin C on hippocampal levels of (I) p16 and (J) p21. Data are expressed as mean ± SD. In panels (B–E), (I), and (J), *p < 0.05, **p < 0.01, ***p < 0.001, ****p < 0.0001, versus CTRL‐Sham; ^##^ *p* < 0.01, ^###^ *p* < 0.001, ^####^ *p* < 0.001, versus WIRS‐Sham; ^$$^ *p* < 0.01, ^$$$$^ *p* < 0.0001, versus WIRS‐SNS. In panels (G) and (H), ****p < 0.0001. n.s.: not significant. Sample size = 3 mice per group.

**Figure S5:** Vitamin C alleviates depressive‐like behavior but fails to restore memory function in WIRS mice. (A) Quantitative results of the total travel distance in the OFT. (B) Quantitative results of the travel distance in the central zone in the OFT. (C) Quantitative results of time spent in the central zone in the OFT. (D) Quantitative results of latency to first enter the central zone in the OFT. (E) Quantitative results of the time fraction of immobility in the FST. (F) Quantitative results of time fraction of immobility in the TST. (G) Quantitative results of the percentage of time spent in the novel arm in the Y‐maze test. (H) Quantitative results of the discrimination index in OLRT. Data are expressed as mean ± SD. *p < 0.05, **p < 0.01, ***p < 0.001, ****p < 0.0001, versus CTRL‐Sham; ^##^ *p* < 0.01, ^###^ *p* < 0.001, versus WIRS‐Sham; ^$^ *p* < 0.05, ^$$$^ *p* < 0.001, versus WIRS‐SNS. Sample size = 4–5 mice per group.

**Figure S6:** Vitamin C fails to restore the hippocampal GFAP expression in WIRS mice. (A) Representative micrographs of immunofluorescence staining for GFAP (red) in the hippoca4mpus and its subregions with DAPI (blue). Scale bar = 250 μm. (B–E) Quantitative results of the relative area fraction of GFAP^+^ signaling in the (B) whole hippocampus, (C) CA1, (D) CA2/3, and (E) DG. Data are expressed as the mean ± SD. *p < 0.05, **p < 0.01, ****p < 0.0001, versus CTRL‐Sham; ^#^ *p* < 0.05, ^###^ *p* < 0.001, versus WIRS‐Sham; ^$$$^ *p* < 0.001, versus WIRS‐SNS. Sample size = 3 mice per group.

## Data Availability

The data that support the findings of this study are available from the corresponding author upon reasonable request.
