# Supplementary figures and images for: Sciatic Nerve Stimulation Mitigates Depression‐Like Behaviors and Memory Deficits in Stressed Mice
Source: Kaohsiung J Med Sci. 2025 Aug 26;42(1):e70091. doi: 10.1002/kjm2.70091 (PMC12782250; doi:10.1002/kjm2.70091)

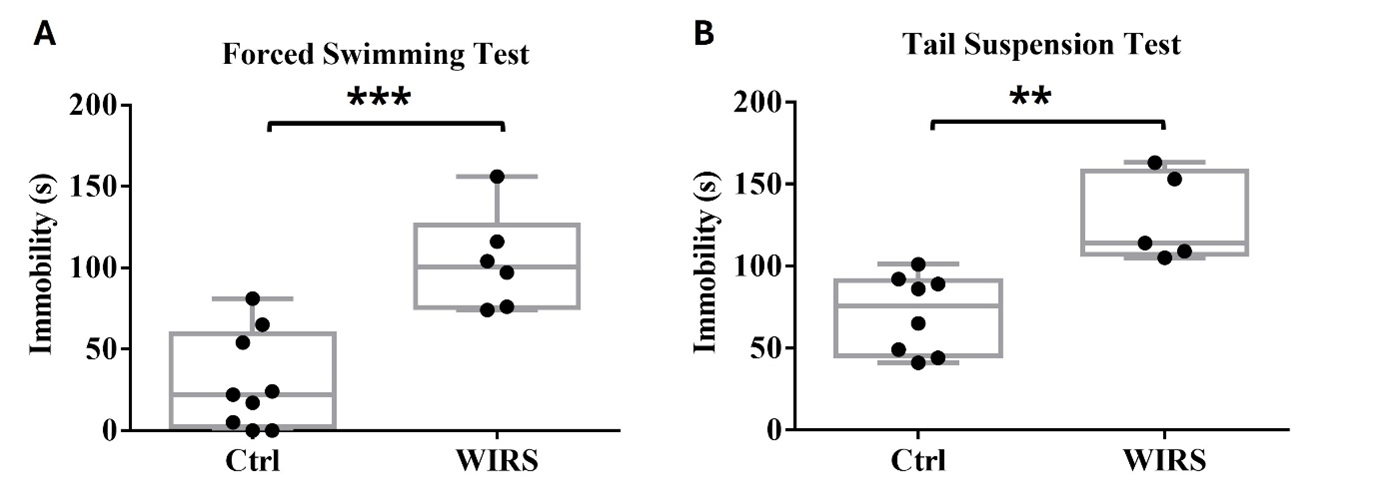

Supplement: Supplementary file 1 — Figure S1: Water Immersion Restraint Stress induced depression‐like behavior in a rodent model. Representative (A) WIRS‐treated mice showed significant immobility compared with nonstressed control mice, as suggested by the Forced Swimming Test (control groups: n = 9; WIRS groups: n = 6). (B) WIRS‐treated mice showed significant immobility compared with nonstressed control mice, as suggested by the Tail Suspension Test (control groups: n = 8; WIRS groups: n = 5). Data are expressed as mean ± SD. *p < 0.05, **p < 0.01, ***p < 0.001. [file KJM2-42-e70091-s006.tif]

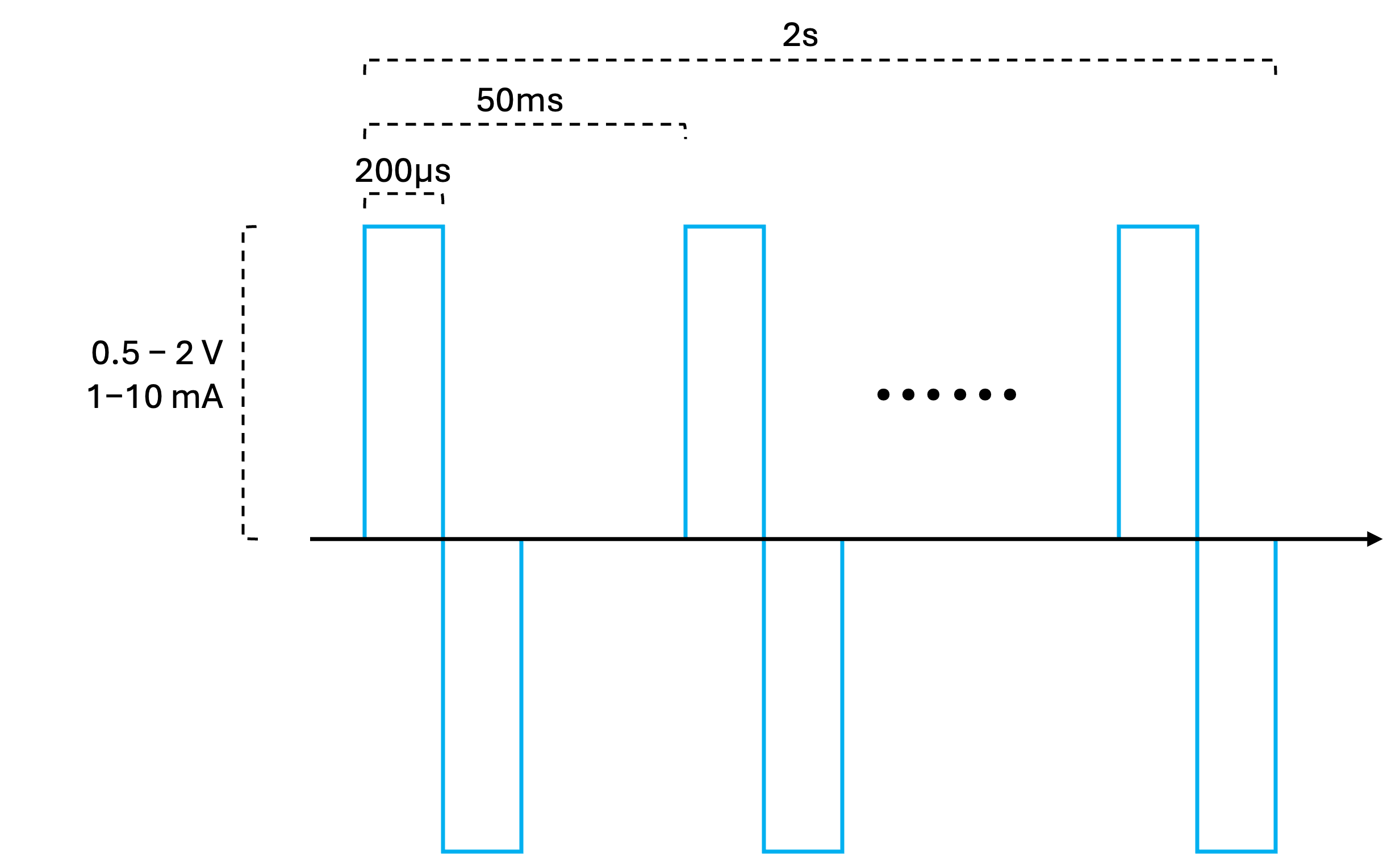

Supplement: Supplementary file 2 — Figure S2: Parameters of the SNS waveform. [file KJM2-42-e70091-s001.tiff]

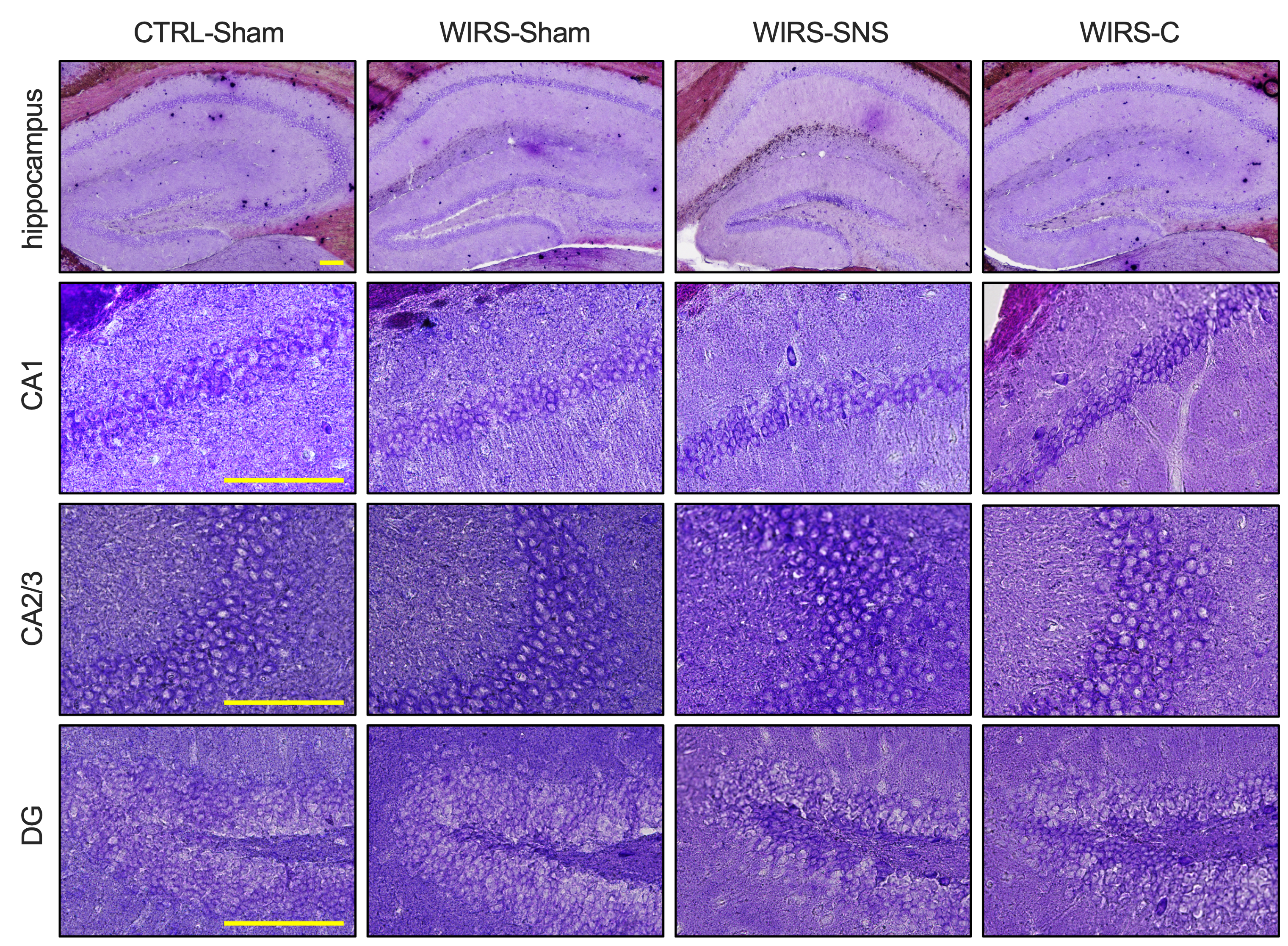

Supplement: Supplementary file 3 — Figure S3: Neither WIRS nor SNS lead to an obvious neuronal loss in the hippocampus of mice. The representative Nissl staining images. Scale: 250 μm. [file KJM2-42-e70091-s004.tiff]

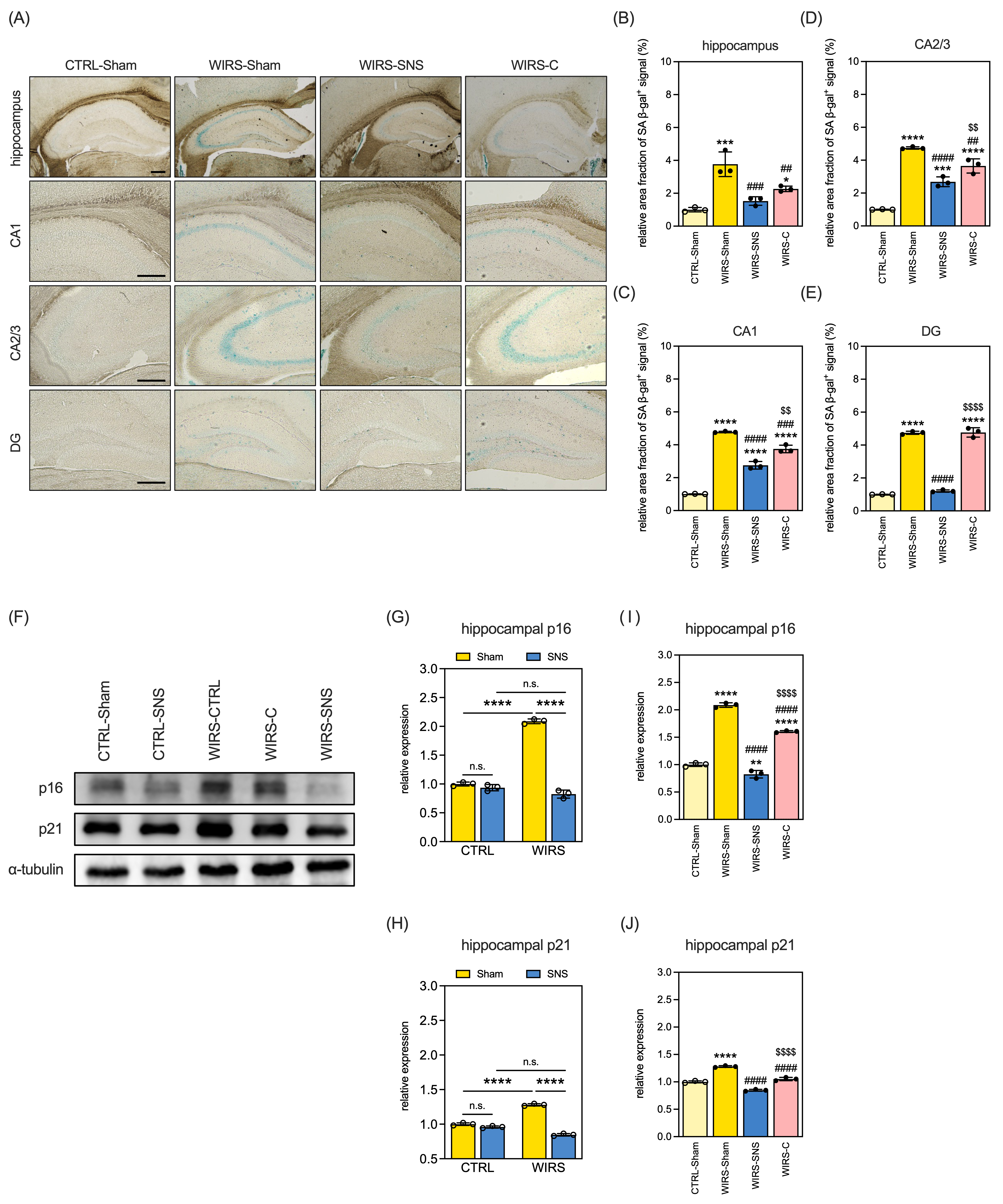

Supplement: Supplementary file 4 — Figure S4: Vitamin C attenuates hippocampal cellular senescence in WIRS mice. (A) Representative micrographs of SA‐β‐gal staining in the hippocampus and its subregions. Scale bar = 250 μm; (B–E) Quantitative results of relative area fraction of SA‐β‐gal+ signaling in the (B) whole hippocampus, (C) CA1, (D) CA2/3, and (E) DG. (F) Representative Western blot images showing hippocampal expression of senescence markers p16 and p21. (G, H) Quantification of the effects of WIRS and SNS on hippocampal levels of (G) p16 and (H) p21. (I, J) Quantification of the effects of WIRS, SNS, and vitamin C on hippocampal levels of (I) p16 and (J) p21. Data are expressed as mean ± SD. In panels (B–E), (I), and (J), *p < 0.05, **p < 0.01, ***p < 0.001, ****p < 0.0001, versus CTRL‐Sham; ## p < 0.01, ### p < 0.001, #### p < 0.001, versus WIRS‐Sham; $$ p < 0.01, $$$$ p < 0.0001, versus WIRS‐SNS. In panels (G) and (H), ****p < 0.0001. n.s.: not significant. Sample size = 3 mice per group. [file KJM2-42-e70091-s002.tiff]

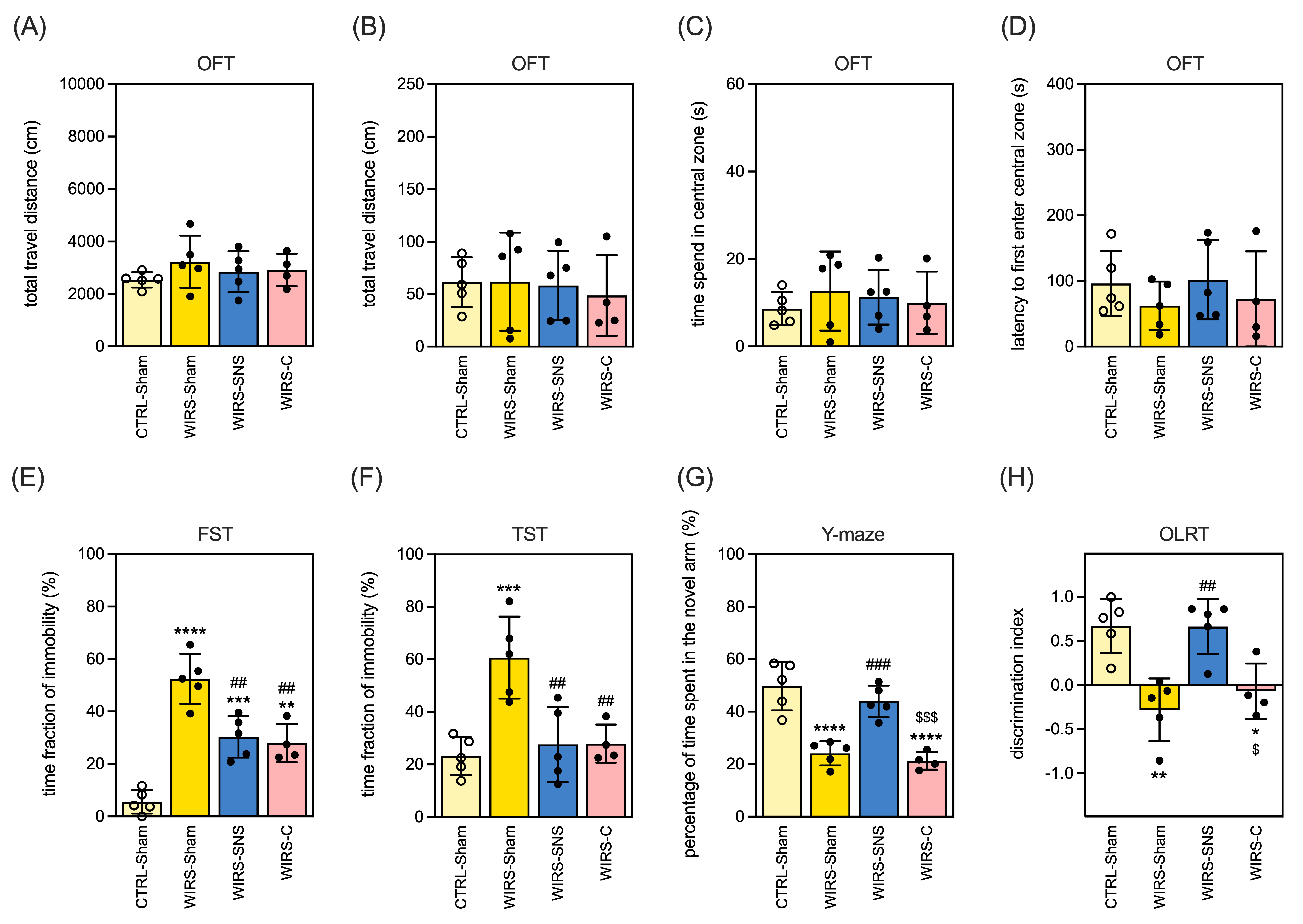

Supplement: Supplementary file 5 — Figure S5: Vitamin C alleviates depressive‐like behavior but fails to restore memory function in WIRS mice. (A) Quantitative results of the total travel distance in the OFT. (B) Quantitative results of the travel distance in the central zone in the OFT. (C) Quantitative results of time spent in the central zone in the OFT. (D) Quantitative results of latency to first enter the central zone in the OFT. (E) Quantitative results of the time fraction of immobility in the FST. (F) Quantitative results of time fraction of immobility in the TST. (G) Quantitative results of the percentage of time spent in the novel arm in the Y‐maze test. (H) Quantitative results of the discrimination index in OLRT. Data are expressed as mean ± SD. *p < 0.05, **p < 0.01, ***p < 0.001, ****p < 0.0001, versus CTRL‐Sham; ## p < 0.01, ### p < 0.001, versus WIRS‐Sham; $ p < 0.05, $$$ p < 0.001, versus WIRS‐SNS. Sample size = 4–5 mice per group. [file KJM2-42-e70091-s005.tiff]

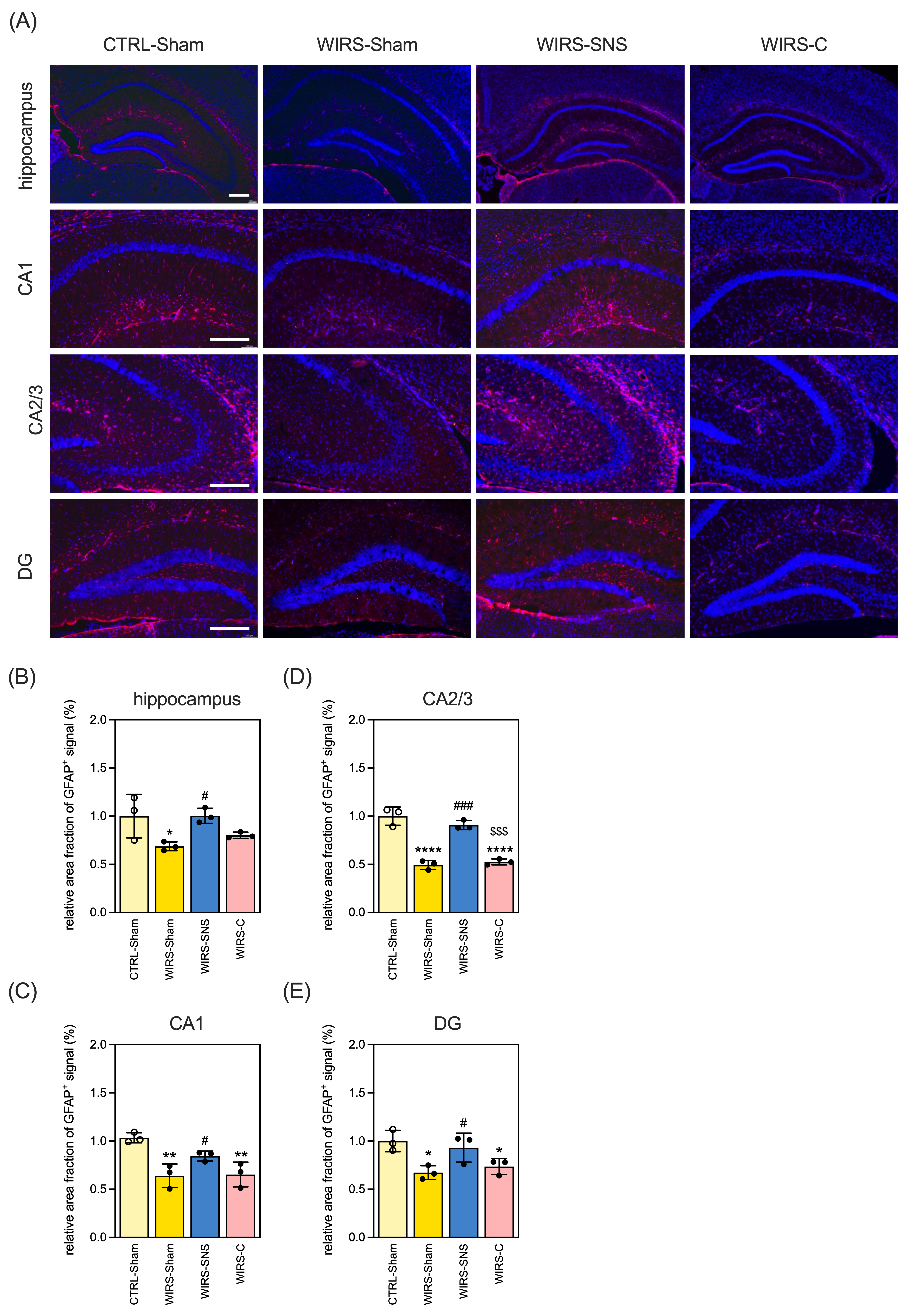

Supplement: Supplementary file 6 — Figure S6: Vitamin C fails to restore the hippocampal GFAP expression in WIRS mice. (A) Representative micrographs of immunofluorescence staining for GFAP (red) in the hippoca4mpus and its subregions with DAPI (blue). Scale bar = 250 μm. (B–E) Quantitative results of the relative area fraction of GFAP+ signaling in the (B) whole hippocampus, (C) CA1, (D) CA2/3, and (E) DG. Data are expressed as the mean ± SD. *p < 0.05, **p < 0.01, ****p < 0.0001, versus CTRL‐Sham; # p < 0.05, ### p < 0.001, versus WIRS‐Sham; $$$ p < 0.001, versus WIRS‐SNS. Sample size = 3 mice per group. [file KJM2-42-e70091-s003.tiff]
